# Simultaneous occurrence of HER2 mutation and PD-L1 high expression in EGFR-mutant NSCLC: a case report

**DOI:** 10.3389/fonc.2026.1953767

**Published:** 2026-10-15

**Authors:** Yunxia Su, Huanzhang Su, Jianguang Lin

**Affiliations:** 1 Department of Medical Oncology, The Second Affiliated Hospital of Fujian Medical University, Quanzhou, Fujian, China; 2 Department of Respiratory and Critical Care Medicine, Quanzhou First Hospital, Quanzhou, Fujian, China

**Keywords:** triple mutation, EGFR-TKIs, PD-L1 high expression, HER2-ADC, trastuzumab rezetecan

## Abstract

In non-small cell lung cancer (NSCLC), EGFR and HER2 mutations are generally considered mutually exclusive events, with a co-occurrence probability estimated to be less than 1%. Patients with either EGFR or HER2 mutations tend to exhibit low PD-L1 expression levels. The simultaneous presence of both EGFR and HER2 mutations accompanied by high PD-L1 expression is exceedingly rare. This study reports a female patient who was admitted to the hospital after a routine physical examination revealed a right lung nodule and was diagnosed with “right lung adenocarcinoma (cT2aN2aM1c1, IVA; EGFR 21 L858R mutation; HER2 exon 8 mutation; PD-L1 TPS 60%).” The patient was immediately started on first-line third-generation EGFR-targeted therapy (Furmonertinib), but disease progression occurred after just 4.4 months (malignant pleural effusion and pericardial effusion). As the patient refused repeat biopsy, treatment with an ADC drug (Trastuzumab rezetecan) was initiated. Disease control was achieved after only one cycle of treatment, and the regimen was continued thereafter. The progression-free survival has exceeded 5.3 months, and at the time of writing this report, the disease remained well-controlled. This paper presents the first report of this rare and therapeutically challenging combination of molecular pathological features—concurrent EGFR and HER2 mutations with high PD-L1 expression in an NSCLC patient. When standard first-line third-generation EGFR-TKIs proved ineffective, the successful use of an anti-HER2 ADC drug to control the tumor demonstrates its potential clinical value. However, whether all patients initially presenting with this pathological molecular subtype will achieve consistent efficacy remains to be further investigated.

## Introduction

In non-small cell lung cancer, EGFR and HER2 mutations are conventionally regarded as mutually exclusive events, with a reported co-occurrence rate of less than 1% (1). Meanwhile, patients harboring either EGFR or HER2 mutations frequently demonstrate low PD-L1 expression. Consequently, the simultaneous presence of EGFR and HER2 mutations accompanied by high PD-L1 expression is exceptionally uncommon (2–4). Reports describing the concurrent presence of all three alterations remain scarce, and the optimal management strategy has yet to be established. Recently, HER2-directed antibody-drug conjugates, particularly Trastuzumab rezetecan, have shown marked antitumor efficacy in HER2-mutant non-small cell lung cancer, positioning them as an important treatment modality for this cohort.

## Case history and examination

A 56-year-old female patient with no history of smoking or family history of malignancy was admitted to the hospital on October 17, 2025, due to a right lung nodule detected during a routine health examination. She had a past medical history of hypertension and diabetes mellitus, with blood pressure and blood glucose levels well controlled, and denied any other significant medical history. Chest CT demonstrated a nodular lesion in the lower lobe of the right lung (3.10 × 1.88 cm), suspected to be malignant, along with enlarged lymph nodes in the ipsilateral hila and mediastinum.

## Methods (differential diagnosis, investigations and treatment)

Given the clinical presentation, Lung cancer was strongly suspected. To confirm the diagnosis, a CT-guided percutaneous biopsy of the right lung mass was performed on October 27, 2025. Pathological findings indicated non-small cell carcinoma, consistent with lung adenocarcinoma based on immunohistochemistry (TTF-1+, CK7+, Napsin-A+, Ki-67 approximately 40%+) (**Figure 1**). To guide subsequent treatment decisions, targeted next-generation sequencing (NGS) based on hybrid capture was performed on the percutaneous lung biopsy specimen with a tumor cell content of approximately 30%. Sequencing data were aligned to the GRCh37/hg19 reference genome. Two somatic missense mutations with definite clinical significance were identified in the same tumor specimen. Specifically, an EGFR exon 21 c.2573T>G (p.L858R) mutation was detected with a variant allele frequency (VAF) of 30.40%, and an activating ERBB2 (HER2) exon 8 c.929C>T (p.S310F) point mutation was identified with a VAF of 25.50%.PD-L1 expression was assessed by immunohistochemistry (IHC) using the Dako 22C3 antibody clone (Agilent Technologies) on the Dako Autostainer Link 48 platform. Both PD-L1 IHC and genetic testing were performed on the identical lung biopsy specimen. The tumor proportion score (TPS) was 60%, with PD-L1 positivity defined as membranous staining in ≥1% of viable tumor cells. Imaging evaluation revealed right hilar and right mediastinal lymphadenopathy, as well as multiple bone metastases. Tumor staging was conducted in accordance with the 9th edition of the IASLC TNM staging system for lung cancer. The final diagnosis was established as right lung adenocarcinoma (cT2aN2aM1c1, stage IVB; EGFR exon 21 L858R mutation; ERBB2 (HER2) exon 8 S310F activating mutation; PD-L1 TPS 60%).

**Figure 1:**
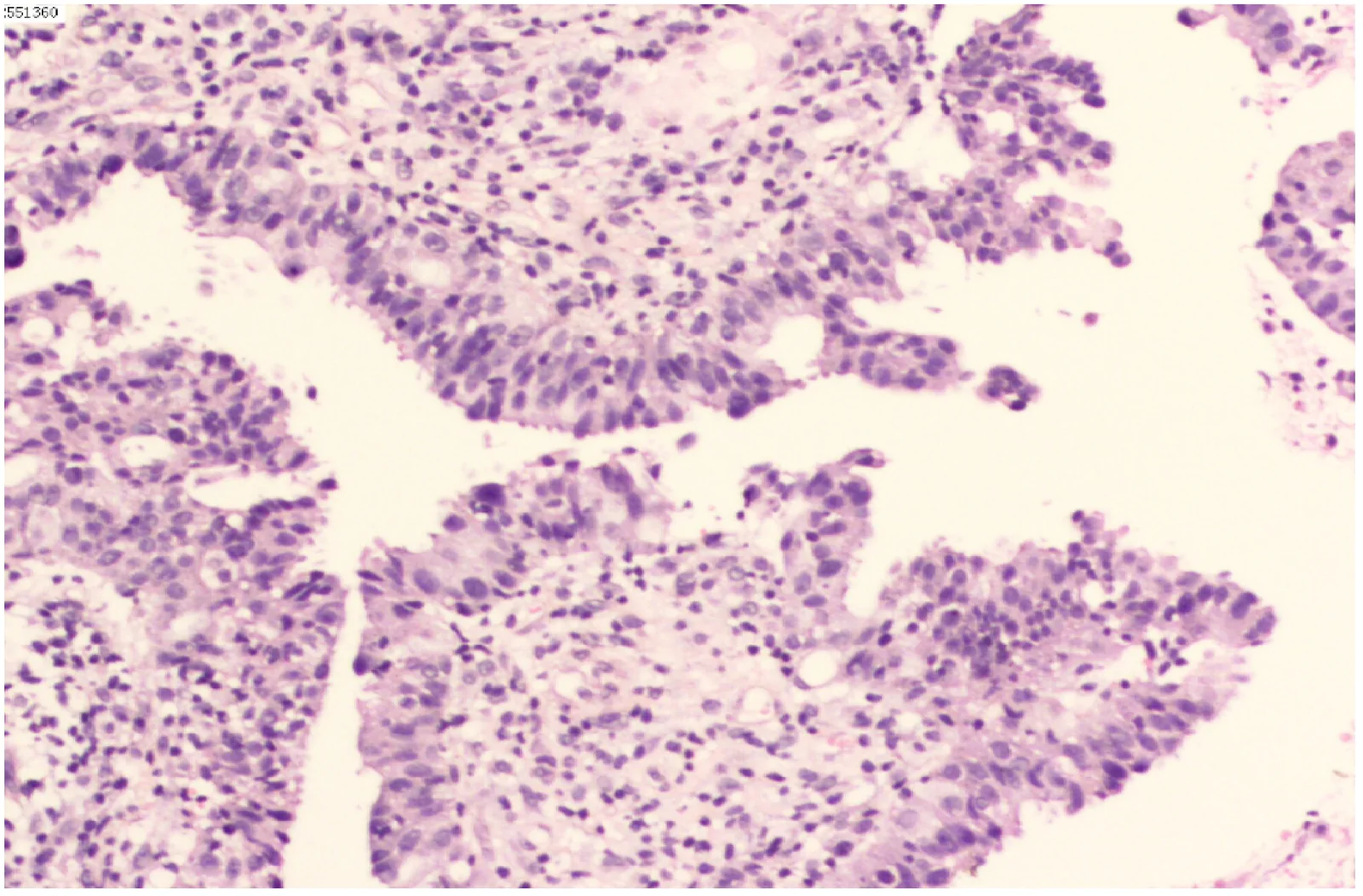
Pathological image of percutaneous lung needle biopsy.

After confirming the diagnosis of lung-cancer through clinical examination and histopathology, a treatment plan was initiated. On November 5, 2025, the patient began first-line targeted therapy with oral Furmonertinib (80 mg once daily), with no significant adverse reactions reported during the treatment period. On March 15, 2026, the patient revisited our hospital due to progressively worsening exertional dyspnea. Chest CT revealed pericardial effusion and bilateral pleural effusion, and echocardiography confirmed large-volume pericardial effusion. Pericardial puncture and drainage were performed on March 18, 2026. On March 23, 2026, follow-up CT revealed a nodular lesion in the lower lobe of the right lung (2.26 × 1.32cm) with a large amount of left-sided pleural effusion, so left-sided thoracentesis and drainage was performed. Cancer cells were detected in both pericardial and pleural effusion specimens, and immunohistochemical staining was suggestive of adenocarcinoma(**Figure 2**). Comprehensive assessment confirmed disease progression, and the patient declined repeat biopsy. On March 27, 2026, second-line targeted therapy with Trastuzumab rezetecan (4.8mg/kg intravenous drip, day 1 every 3 weeks) was initiated.

**Figure 2:**
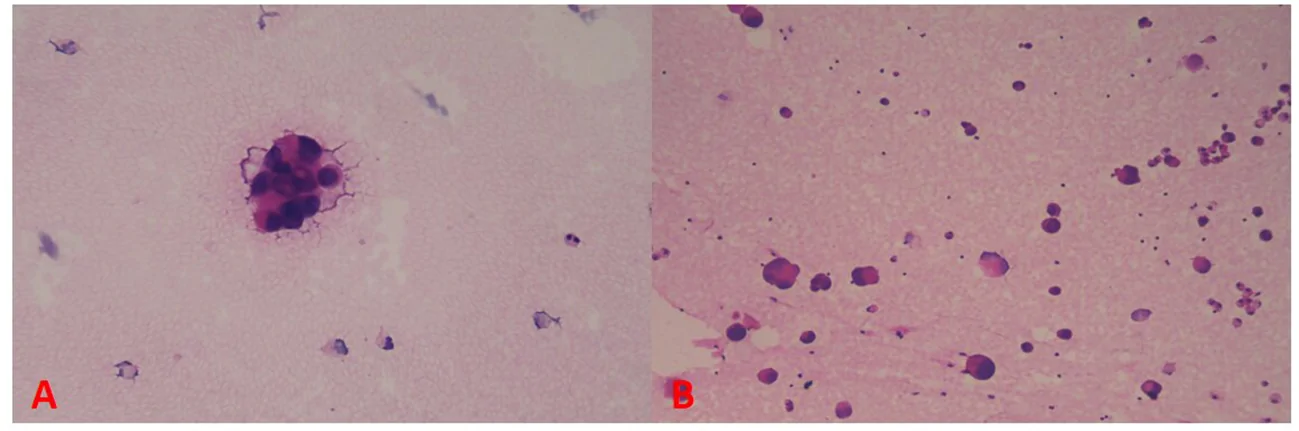
**(A)** Pathological image of pericardiocentesis; **(B)** Pathological image of thoracentesis for pleural effusion.

## Results (outcome and follow-up)

After the 1 cycle of Trastuzumab rezetecan, her dyspnea symptoms improved markedly, and follow-up CT showed tumor shrinkage to 1.9 × 1.27 cm with marked reduction of pleural and pericardial effusions. Subsequently, cycles 2 through 7 were completed as scheduled. Grade III myelosuppression occurred after cycle 2 (white blood cell count 2.18×10^9^/L, neutrophil count 0.9×10^9^/L), and the patient reported no obvious discomfort. Recombinant human granulocyte-colony-stimulating factor (G-CSF, 300 μg daily for two consecutive days) was given. The neutrophil count gradually recovered, and dose modification of Trastuzumab rezetecan was not considered necessary. No further episodes of myelosuppression or other adverse events occurred during subsequent treatment cycles. No adverse events such as interstitial pneumonia were observed. Serial CT on June 03, 2026 demonstrated further tumor shrinkage to 1.43 × 1.20 cm, with near-complete resolution of effusions. The best response was partial response (PR). At the time of data cutoff (September 4, 2026), the patient remained asymptomatic and maintains good performance status, with a PFS of 5.3 months (**Figure 3**).

**Figure 3:**
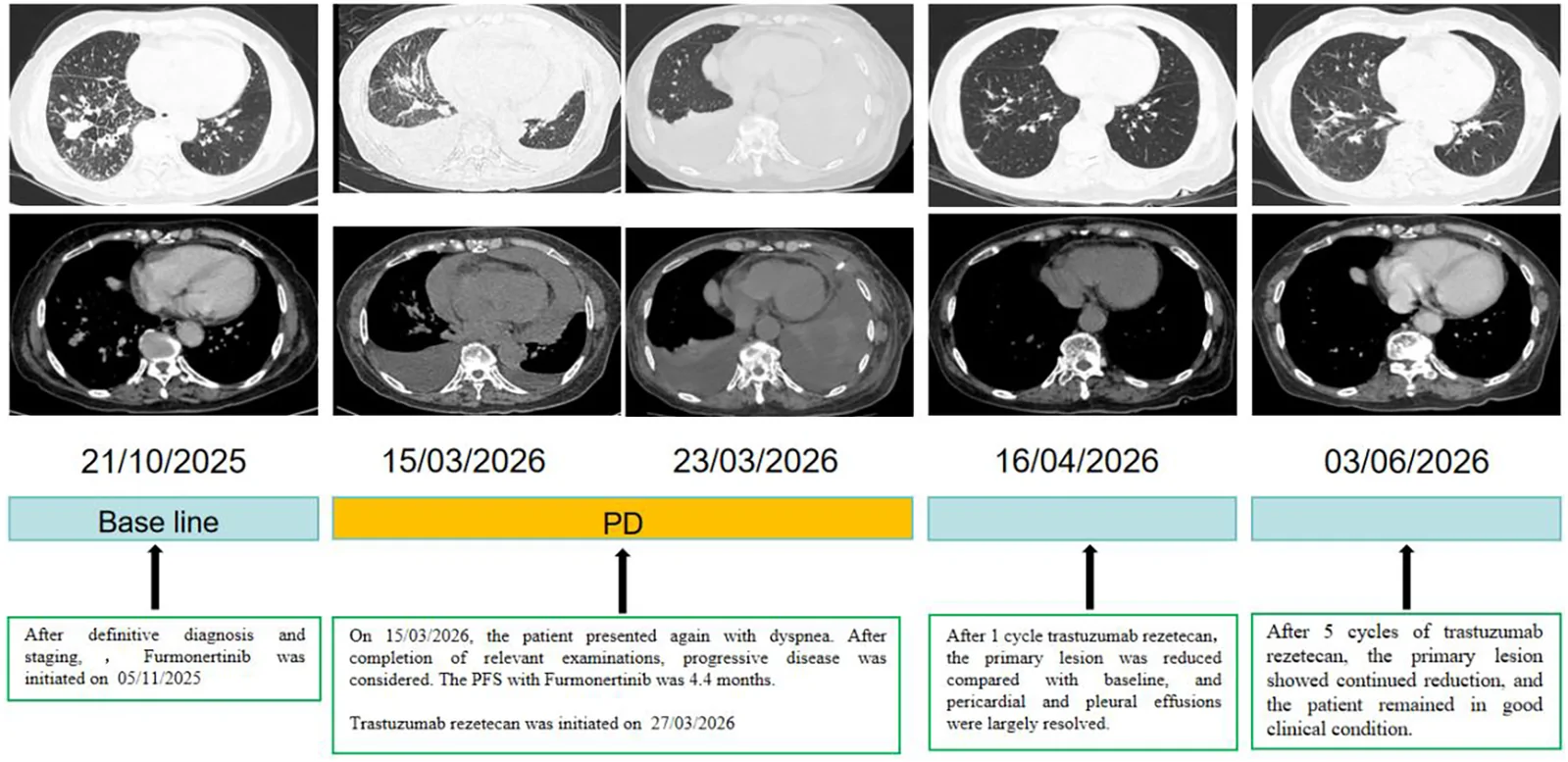
The treatment timeline and clinical response.

## Discussion

This patient concurrently harbored EGFR, HER2 and high PD-L1 expression on tumor cells, representing a rare and therapeutically challenging constellation of molecular pathological features. Studies have suggested that single-agent immunotherapy may have poor efficacy in EGFR-mutant patients with high PD-L1 expression (5). Patients with actionable driver-gene-positive tumors may be at risk of hyperprogressive disease when receiving single-agent immunotherapy (6). In addition, studies suggest that high PD-L1 expression is a potential factor associated with primary resistance to EGFR-TKIs (7). The FLAURA2 trial demonstrated that in patients harboring the EGFR L858R mutation, third-generation EGFR-TKI plus chemotherapy produced a 10.8 month prolongation in PFS relative to the single-agent targeted-therapy group, with a median PFS of 24.7 months (8). Another study reported that EGFR-mutant patients with high PD-L1 expression treated with EGFR-TKI monotherapy had a median PFS of only 7.9 months, which was markedly shorter than that seen in patients with low PD-L1 expression (9). This finding differs substantially from the typical efficacy profile of third-generation EGFR-TKIs; the same study also suggested that chemotherapy combined with EGFR-TKIs may still confer certain clinical benefits, yielding a median PFS of 18.2 months. Accordingly, we initially recommended EGFR-TKI in combination with platinum-based doublet chemotherapy as the firs-line regimen. However, the patient refused chemotherapy and chose Furmonertinib treatment for economic reasons, achieving a PFS of just 4.4 months, which is consistent with the aforementioned findings. Since the patient declined re-biopsy, the exact mechanism underlying primary resistance to EGFR-TKI could not be clarified. Although high PD-L1 expression and concurrent HER2 mutation may serve as potential contributing factors, multiple factors including intrinsic tumor heterogeneity, activation of bypass signaling pathways and host-related factors may jointly shape the therapeutic outcome.

Given that this patient harbors a rare compound mutation and lacks a standard treatment regimen, the choice of treatment after disease progression posed a significant clinical challenge. The HORIZON-Lung study enrolled 94 patients with HER2-mutant non-small-cell lung cancer who had previously received platinum-based chemotherapy plus anti-PD-1/anti-PD-L1 therapy, including three patients harboring the HER2 p.S310F mutation, the study data revealed that the objective response rate (ORR) of Trastuzumab rezetecan in the second-line setting for non-small-cell lung cancer was 74.5%, with a median progression-free survival (PFS) of 11.5 months (10, 11). Based on the proven efficacy of Trastuzumab rezetecan in HER2-mutant NSCLC, the patient was treated with second-line Trastuzumab rezetecan. After a single cycle, a partial response was achieved, and her PFS has extended beyond 5.3 months at the time of this report (September 4, 2026).

The mechanism of action of Trastuzumab rezetecan involves blockade of the HER2 signaling pathway, exploitation of the intrinsic bystander effect of ADCs, and induction of immunogenic cell death, thereby achieving precise tumor cell killing while promoting tumor antigen release, and effectively inhibiting tumor progression through enhancement of local immune responses. Several current studies have observed that EGFR-mutant patients with high PD-L1 expression exhibit suboptimal efficacy with EGFR-TKI therapy (5–8). Currently, evidence regarding the efficacy of HER2-directed antibody-drug conjugates according to PD-L1 expression status is still scarce. Preliminary exploratory studies of Trastuzumab rezetecan have demonstrated that it maintains antitumor activity in non-small cell lung cancer across varying PD-L1 TPS levels, with its therapeutic effect seemingly independent of PD-L1 expression (12). This may partially explain why the patient in this case had a poor response to EGFR-TKIs yet achieved a remarkable response to Trastuzumab rezetecan.

Meanwhile, HER2 is considered a potential resistance factor to EGFR-targeted therapy. At the time of initial diagnosis, the patient was identified as having coexisting HER2 and EGFR mutations, exhibited a poor response to EGFR-TKI therapy, and subsequently achieved significant clinical remission after switching to anti-HER2 treatment. Response to HER2-targeted ADCs has been observed after progression on EGFR-TKIs, suggesting that HER2 may play an important biological role in this tumor. Nevertheless, whether HER2 serves as the primary oncogenic driver in patients with this molecular subtype remains unclear. Clinical trials of anti-HER2-ADC agents for first-line treatment of treatment-naive advanced non-small-cell lung cancer with HER2 mutations are currently underway, and their data are highly anticipated. Meanwhile, a clinical trial (NCT03944772) has explored the combination of EGFR-TKIs and HER2-ADCs to simultaneously block the EGFR and HER2 signaling pathways, which has demonstrated preliminary anti-tumor activity. Nevertheless, these findings need to be further validated in future prospective studies.

We also recognize that the treatment response observed in this case report for this unique molecular pathological subtype may lack generalizability. In addition, this study has limitations, including the absence of repeat biopsy to clarify resistance mechanisms and the relatively short follow-up duration. In summary, the findings from this report remain to be further validated in subsequent prospective studies.

## Patient perspective informed consent

When I was diagnosed with advanced lung cancer, I felt devastated and frightened. My disease progressed rapidly after first-line Furmonertinib therapy. Massive pleural and pericardial effusions caused severe dyspnea, leaving me unable to lie flat at night, which was even more distressing. My oncology team thoroughly explained the ADC regimen of Trastuzumab rezetecan to me. I experienced barely any obvious discomfort after starting this therapy. To my surprise, I tolerated the treatment well and regained a good quality of life.

## Conclusion

To our knowledge, this case represents the first report of a non-small cell lung cancer patient harboring concurrent EGFR and HER2 mutations along with high PD-L1 expression, who achieved a remarkable response to second-line monotherapy with Trastuzumab rezetecan. It provides important real-world evidence to inform future research.

## Data Availability

The original contributions presented in the study are included in the article/supplementary material. Further inquiries can be directed to the corresponding author.
